# Quirks of track preservation and formation are more likely than pre-avian flight and ultrafast dinosaurs

**DOI:** 10.1073/pnas.2500877122

**Published:** 2025-04-01

**Authors:** Peter L. Falkingham, Jens N. Lallensack

**Affiliations:** ^a^School of Biological and Environmental Sciences, Liverpool John Moores University, Merseyside L3 3AF, United Kingdom; ^b^Departamento de Ciência da Computação, Universidade Federal de Minas Gerais, Belo Horizonte, MG 31270-901, Brazil

That fossil trackways can record behavior of long extinct animals is one of their most enticing aspects. However, it is notoriously difficult to test interpretations. In the best cases, hypotheses of behavior can be supported through observing extant taxa making similar traces. Unfortunately, such extant comparison was not present to support the exceptional claims by Dececchi et al. (1) in their paper arguing that a trackway represented evidence of pre-avian aerial behavior. This omission is particularly pertinent, given the weaknesses of their track-based evidence.

Their conclusions rely on the three tracks identified as “Trackway 2” being a complete and fully preserved trackway. Their argument (in extended methods) is that if trackway 2 were incomplete, trackway 1 would also be incomplete. This presupposes that the trackways were made approximately simultaneously, in sediment of identical consistency, and ignores any effects of either time averaging or spatial heterogeneity of the substrate. Even over periods of hours, a sediment can dry nonuniformly, making a subsequent trackway look different. More obviously, there are no tracks prior to L1 or after L4 of trackway 1, so unless the authors are claiming this represents both landing and take-off (without variation in stride lengths), then we *know* that tracks *are* missing from the surface. Isolated tracks are common on tracksites even where more complete trackways are present.

The evidence for R1, L1, and R2 being part of the same trackway is weak. L1 and R2 are clearly different (their figure 1), in both divarication angle and digit width. Furthermore, the orientation of the three tracks is inconsistent, with L1 pointing away from, and R2 pointing toward, the trackway midline, suggesting that these are isolated tracks. The tracking surface presents other isolated tracks (their figure 1B). Track 3 (R2) of Trackway 2 is also located beside three other impressions (T3, T4, and T7). Two more isolated impressions (T9 and T8) are closer to R2 than L1 and have no associated trackway. Additional tracks T5 and T6 appear on the surface crossing between L1 and R2, with a similarly unusual stride length. The authors did not analyze any of these tracks. Unfortunately, while providing photogrammetric models of individual tracks, the authors did not present a high-resolution model of the surface, or even the trackway, so readers cannot take a closer look.

**Fig. 1.**
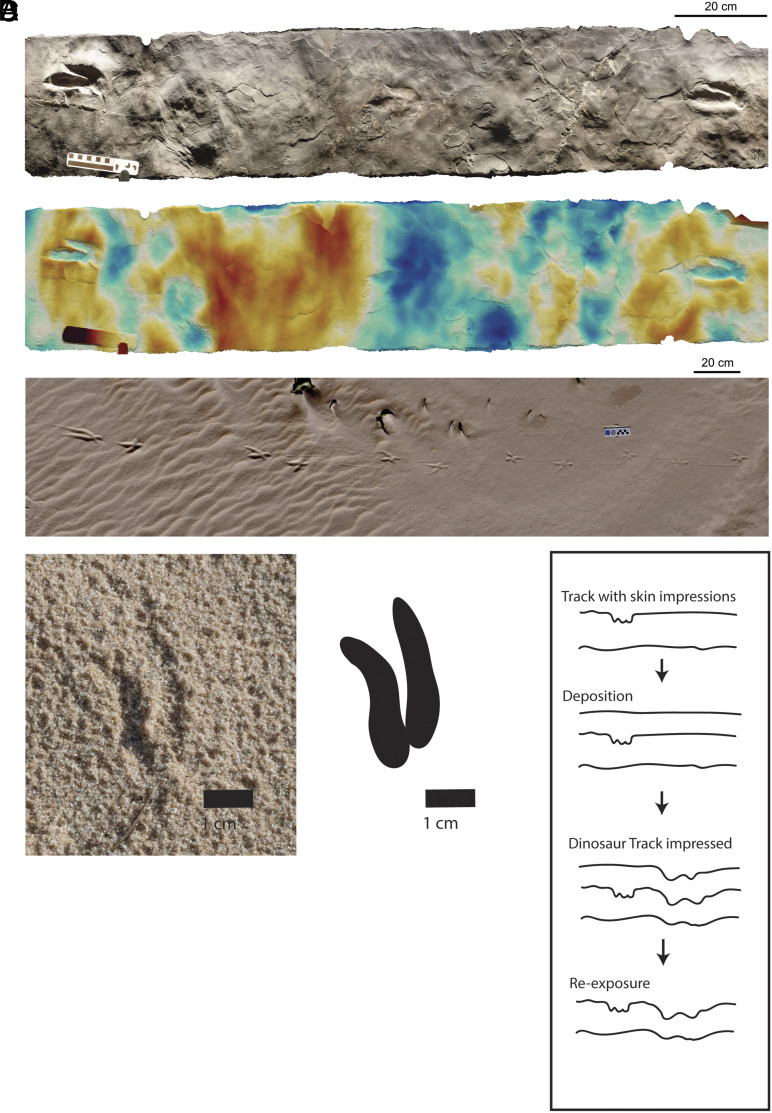
(*A*) Medium-sized theropod trackway from Beneski Museum of Natural History (Specimen 6A) presented as height map, illustrating how tracks can easily be reduced or missing within a trackway, and how tridactyl trackmakers can leave didactyl tracks. (*B*) Take-off trace of a large bird transitioning from a walk to a skip. Note that when legs are involved in take-off, tracks are deeper just before leaving the ground. (*C*) Small pheasant track appearing as an isolated print on a moist sand. Left during normal walking by a tridactyl trackmaker, the morphology and size are very similar to those reported by Dececchi et al. (*D*) One possible explanation for how low-anatomical fidelity dinosaur tracks can appear on the same surface as tracks with skin impressions. This mode of formation and preservation can easily result in some tracks missing from within a trackway if transmission or penetration is uneven along the original trackway.

The authors discount that trackway 2 may represent transmitted or penetrative undertracks because “the Jinju tracksite also has almost perfectly preserved tracks of lizards as well as three-toed *Minisauripus* theropod tracks with perfect skin traces”, which again ignores possible time averaging (Fig. 1*D*). That the tracks of trackway 2 do not preserve skin impressions, if anything, suggests strongly that they were made at a different time or in a different substrate consistency.

It is far more likely that R1, L1, and R2 do not form a continuous and complete trackway, either because they are misattributed or because the trackway is missing tracks, than it is that this animal was moving unreasonably fast for its size and engaging in wing-assisted behavior.
